# Supplementary material for: Towards Understanding Afghanistan Pea Symbiotic Phenotype Through the Molecular Modeling of the Interaction Between LykX-Sym10 Receptor Heterodimer and Nod Factors
Source: Front Plant Sci. 2021 May 7;12:642591. doi: 10.3389/fpls.2021.642591 (PMC8138044; doi:10.3389/fpls.2021.642591)
Supplement: Supplementary file 1 [file Data_Sheet_1.pdf]

Supplementary material

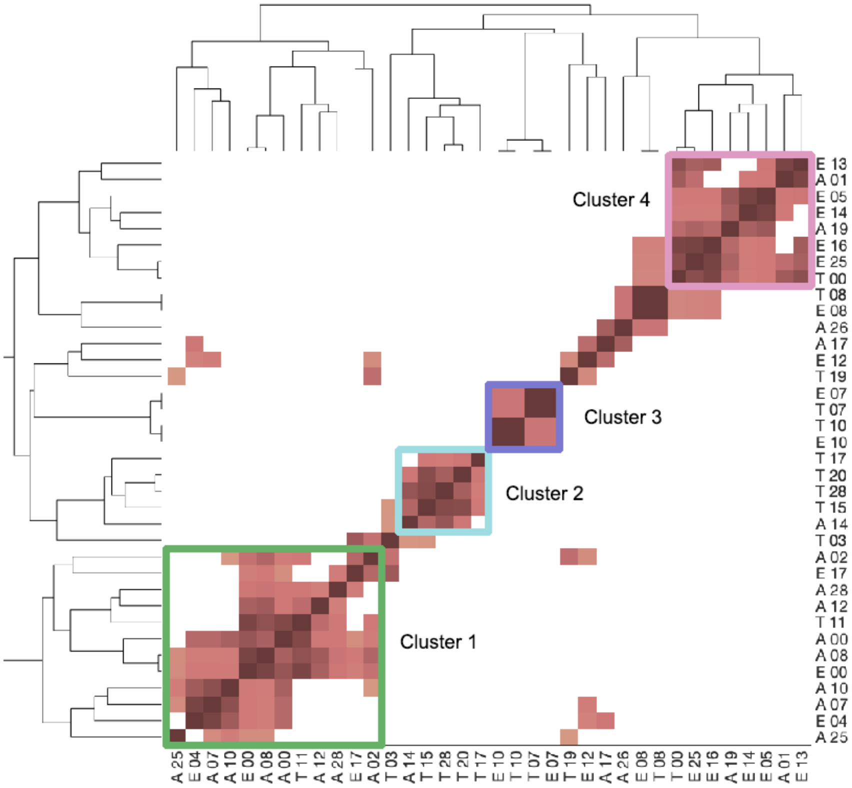

Supplementary Figure 1. Similarity of mutual orientation between LykX and Sym10 receptors in dimers. Four separate clusters were identified and highlighted with colors. Afghan alleles are denoted with A, Tajik – with T, and European – with E.

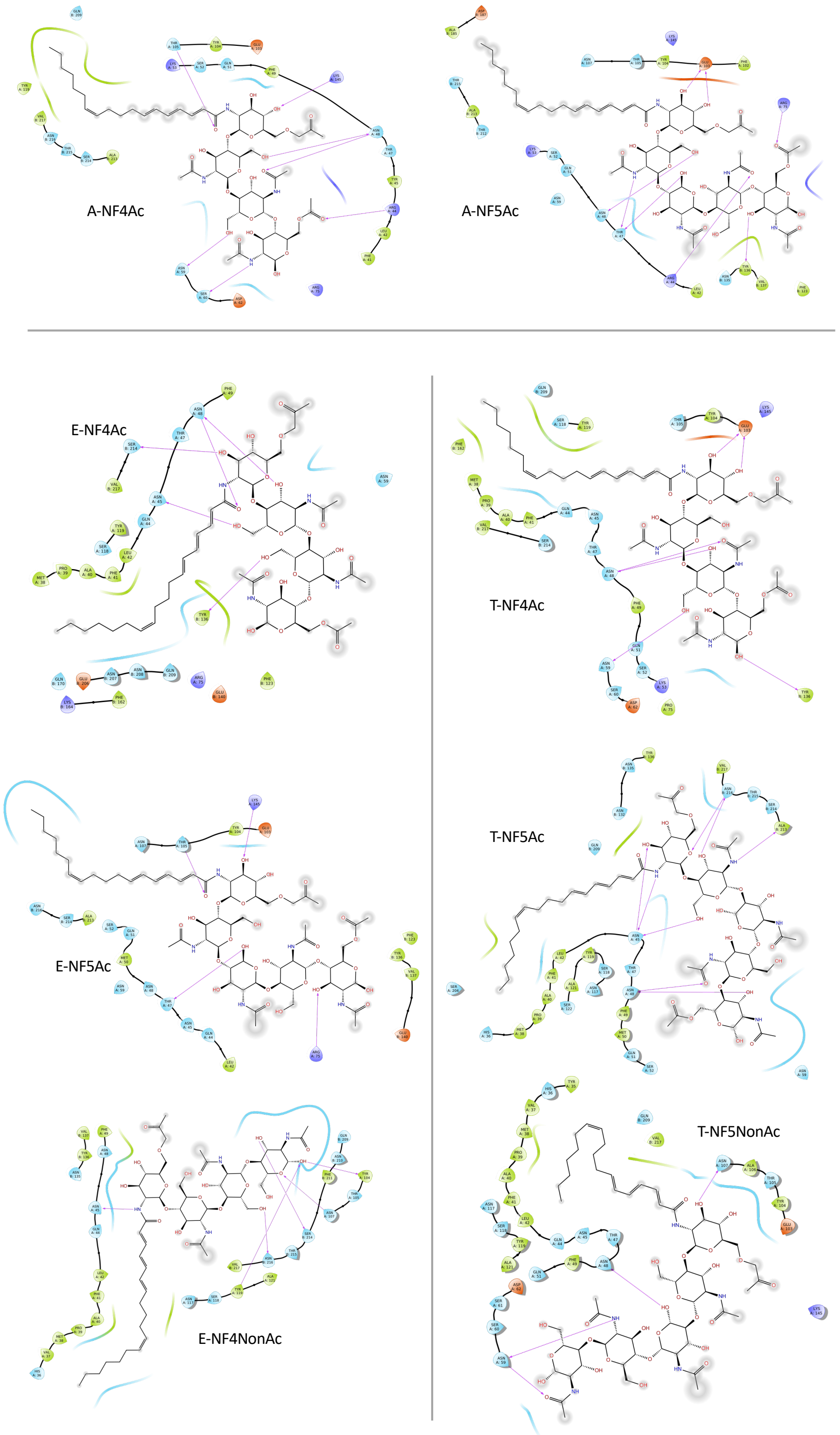

Supplementary Figure 2. Protein-ligand interactions in 8 LyxX-Sym10-NFs complexes. Hydrogen bonds are marked as purple arrow lines.

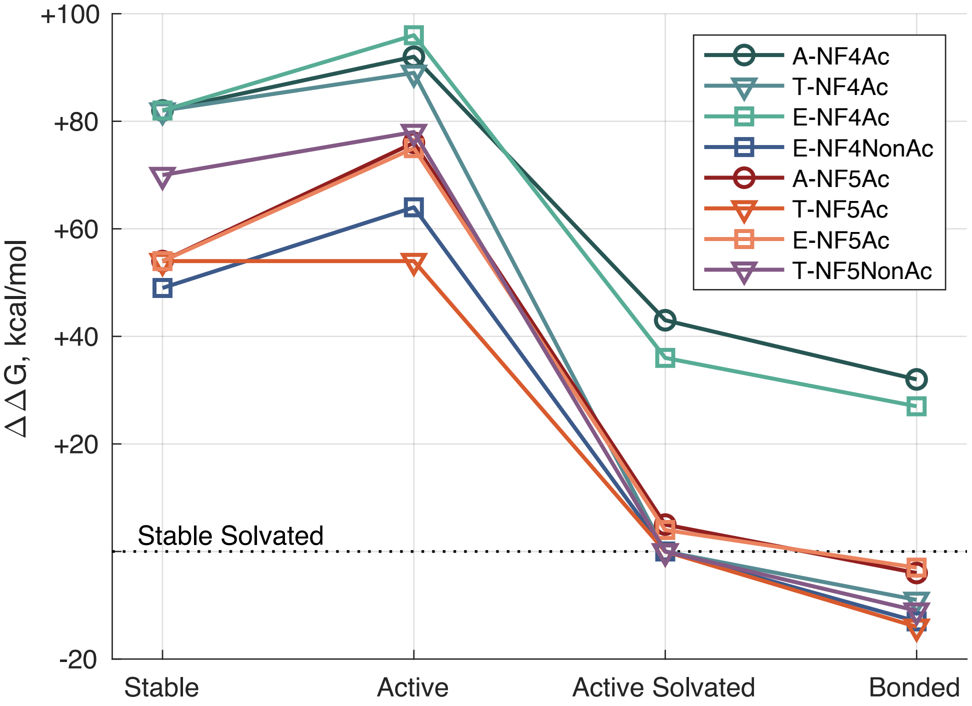

Supplementary Figure 3. *ΔΔ*G relative free energy plot for LykX-Sym10- NFs complexes in different computational conditions. The 0-value at Y-axis corresponds to the Stable Solvated energy as a reference level. Energies in other conditions are presented as deviations from the reference.

Supplementary Table 1. *P. sativum* receptor genes annotation

Receptor gene	Allele	Accession numbers	References	Amount
PsSym10				*8*
	EU	AJ575250	Madsen et al., 2003	
	EU	AJ575251	Madsen et al., 2003	
	EU	AJ575252	Madsen et al., 2003	
	EU	AJ575253	Madsen et al., 2003	
	EU	MN727811	This work	
	AF	MN727808	This work	
	AF	MN727809	This work	
	AF	MN727810	This work	
PsLykX				*95*
	EU	MF155382-MF155468	Sulima et al., 2017	*87*
	AF	MN187362	Sulima et al., 2019	
	TJ	MN187363	Sulima et al., 2019	
	AF	MN200353	Sulima et al., 2019	
	AF	MN200354	Sulima et al., 2019	
	AF	MN200355	Sulima et al., 2019	
	TJ	MN200356	Sulima et al., 2019	
	AF	MN200357	Sulima et al., 2019	
	AF	MN200358	Sulima et al., 2019	

Supplementary Table 2. Primers used for sequencing of PsSym10 alleles (5'-3') and corresponding temperature.

Sym10 fw1 (58°C)	CTT GCA TTT CTT CAC AAT TTC AC
Sym10 fw2 (58°C)	TAA GTC CAA ATC TAT TGC CAC C
Sym10 fw3 (59°C)	CAA GGC TAA TAT AGA TGG TAG AG
Sym10 fw4 (58°C)	TGA TTG AGT TGC TTA CCG GC
Sym10 fw5 (58°C)	ACA TAC TTT GCT CGG TCT CC
Sym10 fw6 (58°C)	GGA TTC ACC ACC TTC ATG TG
Sym10 rv1 (58°C)	GTA AGA CTT GGC CTT CAA CC
Sym10 rv2 (58)	CCG ATT TTG ATC CCT GTG AC
Sym10 rv3 (58°C)	GTA AGC GAG ACC ACC GAG
Sym10 rv4 (58°C)	CAA CTA AGA GCA TTA TCA ATA GG
Sym10 rv5 (58°C)	ACC ATA TAG TTC CAC ACA TAA AG
Sym10 rv6 (59°C)	CTT CCA TTT GAA GAT GGT TGA TC
Sym10 rv7 (58°C)	GCT AAT ACC AAT TAT AAA AGC AGG

Supplementary Table 3. Amino acid polymorphism in the LykX protein of the European, Tajik, and Afghan subpopulations. In positions #44, #45, #75, and #76 subpopulation-specific differences are marked by bold, other variable amino acids in polymorphic sites are separated by '~' symbol. In position #45 both asparagine (N) and serine (S) are relevant for European subpopulation, but serine was presented only in the single sequence.

Index	Number of position	Domain	Variable amino acids			Blosum 90 matrix
			Afghanistan	Tajik	European	
1	5	Signal peptide	F	F	F ~ L	0
2	9	Signal peptide	L	L	F ~ L	0
3	13	Signal peptide	L	L	V ~ L	4
4	14	Signal peptide	E	E ~ D	E	3
5	16	Signal peptide	V	V	V ~ F	-1
6	18	Signal peptide	F	F	S ~ F	-3
7	23	Unstructured region	K	K	K ~ Q	1
8	42	LysM1	K	K	K ~ L	-3
9	44	LysM1	R	Q	Q	1
10	45	LysM1	Y	N	N ~ S	-5 / -3
11	48	LysM1	N	N	N ~ K	0
12	75	LysM1	R	P	R	-2
13	76	LysM1	D	A	A	-3
14	82	LysM1	F	F	S ~ F	-3
15	86	LysM1	I	I	V ~ I	5
16	111	LysM2	T	T	T ~ S	3
17	128	LysM2	V	V ~ F	V	-1
18	134	LysM2	S	S	G ~ S	1
19	136	LysM2	H	H	H ~ D	-4
20	142	LysM2	I	A ~ I	A ~ I	-2
21	183	LysM3	A	A	A ~ I	-2
22	184	LysM3	S	S	S ~ F	-3
23	191	LysM3	I	I	M ~ I	3

Supplementary Table 4. Protein amino acid sequences similarity in percent (%) between three model proteins and four *P. sativum* model proteins. The 5LS2 and the 4EBZ sequences are significantly closer to each other and to target proteins than the 5JCD sequence. The highest sequence similarity with target proteins was detected for the 5LS2 model, its values are marked by bold.

Model	4EBZ	5JCD	5LS2
Afghanistan_Sym10	23.6%	18.7%	**23.9%**
European_Sym10	22.9%	18.3%	**24.4%**
Afghanistan_LykX	45.2%	18.0%	**49.5%**
European_LykX	44.7%	18.6%	**48.9%**

Supplementary Table 5. The most crucial amino acids involved in dimer-NF interactions during MD simulations. Data is presented as percentage of trajectory duration.

Amino acid	A-NF4Ac	A-NF5Ac	E-NF4Ac	E-NF4NonAc	E-NF5Ac	T-NF4Ac	T-NF5Ac	T-NF5NonAc
LykX_103	94%	14%	0%	2%	70%	86%	0%	61%
LykX_104	13%	27%	34%	22%	24%	23%	8%	24%
LykX_105	80%	52%	23%	34%	99%	95%	8%	97%
LykX_107	1%	38%	38%	44%	0%	0%	10%	61%
LykX_119	17%	30%	20%	11%	25%	15%	24%	59%
LykX_40	2%	2%	62%	9%	2%	2%	68%	0%
LykX_41	29%	22%	33%	40%	24%	31%	14%	45%
LykX_42	0%	0%	22%	31%	23%	1%	31%	0%
LykX_44	39%	8%	24%	6%	42%	83%	19%	8%
LykX_45	19%	56%	88%	67%	100%	75%	75%	7%
LykX_47	65%	8%	4%	1%	77%	64%	0%	71%
LykX_48	94%	77%	81%	55%	72%	78%	20%	81%
LykX_49	31%	32%	2%	46%	9%	28%	4%	22%
LykX_52	77%	2%	0%	0%	50%	86%	0%	55%
LykX_59	45%	1%	1%	0%	12%	61%	1%	71%
Sym10_132	0%	16%	0%	40%	62%	0%	14%	0%
Sym10_135	0%	0%	11%	68%	41%	8%	6%	0%
Sym10_136	2%	1%	65%	49%	74%	17%	56%	0%
Sym10_137	0%	0%	53%	16%	35%	12%	0%	0%
Sym10_211	18%	0%	24%	43%	0%	2%	4%	46%
Sym10_213	9%	0%	29%	68%	4%	0%	43%	3%
Sym10_214	0%	0%	82%	71%	52%	0%	24%	2%
Sym10_215	0%	32%	6%	46%	6%	0%	26%	0%
Sym10_216	0%	74%	57%	61%	81%	0%	64%	0%
Sym10_217	2%	18%	21%	8%	17%	2%	39%	2%
