# Supplementary material for: Towards Understanding Afghanistan Pea Symbiotic Phenotype Through the Molecular Modeling of the Interaction Between LykX-Sym10 Receptor Heterodimer and Nod Factors
Source: Front Plant Sci. 2021 May 7;12:642591. doi: 10.3389/fpls.2021.642591 (PMC8138044; doi:10.3389/fpls.2021.642591)
Supplement: Supplementary file 2 [file Data_Sheet_2.ZIP › LjNFR1.html]

xml version="1.0"?jp\_OjJFRGy/1-621Lupas\_21Lupas\_14Lupas\_28jnetpredJNETCONFJNETSOL25JNETSOL5JNETSOL0JNETHMMJNETPSSMJNETJURY

xml version="1.0"?102030405060708090100110120130140150160170180190200210220230240250260270280290300310320330340350360370380390400410420430440450460470480490500510520530540550560570580590600610MKLKTGLLLFFILLLGHVCFHVESNCLKGCDLALASYYILPGVFILQNITTFMQSEIVSSNDAITSYNKDKILNDINIQSFQRLNIPFPCDCIGGEFLGHVFEYSASKGDTYETIANLYYANLTTVDLLKRFNSYDPKNIPVNAKVNVTVNCSCGNSQVSKDYGLFITYPIRPGDTLQDIANQSSLDAGLIQSFNPSVNFSKDSGIAFIPGRYKNGVYVPLYHRTAGLASGAAVGISIAGTFVLLLLAFCMYVRYQKKEEEKAKLPTDISMALSTQDASSSAEYETSGSSGPGTASATGLTSIMVAKSMEFSYQELAKATNNFSLDNKIGQGGFGAVYYAELRGKKTAIKKMDVQASTEFLCELKVLTHVHHLNLVRLIGYCVEGSLFLVYEHIDNGNLGQYLHGSGKEPLPWSSRVQIALDAARGLEYIHEHTVPVYIHRDVKSANILIDKNLRGKVADFGLTKLIEVGNSTLQTRLVGTFGYMPPEYAQYGDISPKIDVYAFGVVLFELISAKNAVLKTGELVAESKGLVALFEEALNKSDPCDALRKLVDPRLGENYPIDSVLKIAQLGRACTRDNPLLRPSMRSLVVALMTLSSLTEDCDDESSYESQTLINLLSVR---------------------------------------------------------------------------------------------------------------------------------------------------------------------------------------------------------------------------------------------------------------------------------------------------------------------------------------------------------------------------------------------------------------------------------------------------------------------------------------------------------------------------------------------------------------------------------------------------------------------------------------------------------------------------------------------------------------------------------------------------------------------------------------------------------------------------------------------------------------------------------------------------------------------------------------------------------------------------------------------------------------------------------------------------------------------------------------------------------------------------------------------------------------------------------------------------------------------------------------------------------------------------------------------------------------------------------------------------------------------------------------------------------------------------------------------------------------------------------------------------------------------------------------------------------------------------------------------------------------------------------------------------------------------------------------------------------------------------------------------------------------------------------------------------------------------------------------------------------------------------------------------------------------------------------------999727999999999871267777777441001348862688751889999992134676431125667777777622777506888615777764221236346447873578888751788037888760367877646774488988436777767777506888873268746888874266620032067777777777602311677767777777777777777765510138999999999998731135566777777777777777777777777777777777777777777777763353688988623777665121047855899985561899984275436888888777652166530030234437855899861577776677777777776428999999999999998516888616774367766566677633414677765677777347888643467765111016646776321222320111321677777777777777302899999986157321188775167888762789999999999862478887764289999993023677777777777640322313799------BBBB-BB-BBBBBB-B---B---B-BBBBBBBB---B-B--BBBBBB-BB-------BB-BB---B-----B-----B-BBBBB-B--B-BBBB-B-B-B----BB--BB--BB--BBBB-BB--BB-B----B---B-BBBBBBBBB----B---B-BBBBBBB----BB--BB--B-B---BBB-BB-BB-BB--BBBBBBBB-B----BB---B---------BBBBBBBBBBBBB-BBBBBBBBB-----------------BBB---B----B---------------B--B----B--BBB--B--BB--B---BBBB-BBBBBBBBB-B----BBBB-B-------BB-BB-BBB-B-B-BBB-BBBBBB---BBBBBBBB---BB--BBB------B-B--BB-BBBBBB-BBBBBB--B---BBBBBBBBBBBBB---B-BBBBBBBBB-BB------BBBBBBBBBBBBBBBBBB-B-BB--BBBBBBBBBBBBBBBB--BB-----------BBB-BBB-BB--------B--BBB--B---B---BBB-BB-BBBBBB------BB-B--BB-BB--B--B---B----------B----------------------B--B----------B-BBBBB------------BB-BB----------B---------------------B-B-B------------------------BB-----BBB---BB----------B-----B-B-B-B------------BBB-B-B-----B--B----------B--B-----------BB----------------------------B----BB-B-----------------------------------------------------------------------B--BB--------------B-BB-B------BBB--B-------B---B--BB---B--BB-B-BBB-----BBBB-BB----B---B------------BB-BB--BB-BB--B-------BB---B----B-------B-BB-B-BB-----------B-B-BB-BBBBBBB-----B---B---B-B-BBBBBB------------------BB--B--BB--------B--BBB--B--------B--BB-BBB-BB---------B--BB--B-------------------------------------------------------------B---------------------------------------------------B----------------------------B-------------B------------------B-B----------------B----------------------------------------------------------------------------------------------------------------------------------------------------B------------------------------BBB--------------B----------B----B-B------BB-----------------------------B--BB-B---B----------------------------------------------------B----------------------B--B-------------------------B-----------------------------B--BB-BB--B-------------B--------------------------------\*\*\*\*\*\*\*\*\*\*\*\*\*\*\*\*\*\*\*\*\*\*\*\*\*\*\*\*\*\*\*\*\*\*\*\*\*\*\*\*\*\*\*\*\*\*\*\*\*\*\*\*\*\*\*\*\*\*\*\*\*\*\*\*\*\*\*\*\*\*\*\*\*\*\*\*\*\*\*\*\*\*
