# Supplementary material for: Towards Understanding Afghanistan Pea Symbiotic Phenotype Through the Molecular Modeling of the Interaction Between LykX-Sym10 Receptor Heterodimer and Nod Factors
Source: Front Plant Sci. 2021 May 7;12:642591. doi: 10.3389/fpls.2021.642591 (PMC8138044; doi:10.3389/fpls.2021.642591)
Supplement: Supplementary file 2 [file Data_Sheet_2.ZIP › LjNFR5.html]

xml version="1.0"?jp\_9ZYOxVh/1-595Lupas\_21Lupas\_14Lupas\_28jnetpredJNETCONFJNETSOL25JNETSOL5JNETSOL0JNETHMMJNETPSSMJNETJURY

xml version="1.0"?102030405060708090100110120130140150160170180190200210220230240250260270280290300310320330340350360370380390400410420430440450460470480490500510520530540550560570580590MAVFFLTSGSLSLFLALTLLFTNIAARSEKISGPDFSCPVDSPPSCETYVTYTAQSPNLLSLTNISDIFDISPLSIARASNIDAGKDKLVPGQVLLVPVTCGCAGNHSSANTSYQIQLGDSYDFVATTLYENLTNWNIVQASNPGVNPYLLPERVKVVFPLFCRCPSKNQLNKGIQYLITYVWKPNDNVSLVSAKFGASPADILTENRYGQDFTAATNLPILIPVTQLPELTQPSSNGRKSSIHLLVILGITLGCTLLTAVLTGTLVYVYCRRKKALNRTASSAETADKLLSGVSGYVSKPNVYEIDEIMEATKDFSDECKVGESVYKANIEGRVVAVKKIKEGGANEELKILQKVNHGNLVKLMGVSSGYDGNCFLVYEYAENGSLAEWLFSKSSGTPNSLTWSQRISIAVDVAVGLQYMHEHTYPRIIHRDITTSNILLDSNFKAKIANFAMARTSTNPMMPKIDVFAFGVLLIELLTGRKAMTTKENGEVVMLWKDMWEIFDIEENREERIRKWMDPNLESFYHIDNALSLASLAVNCTADKSLSRPSMAEIVLSLSFLTQQSSNPTLERSLTSSGLDVEDDAHIVTSITAR---------------------------------------------------------------------------------------------------------------------------------------------------------------------------------------------------------------------------------------------------------------------------------------------------------------------------------------------------------------------------------------------------------------------------------------------------------------------------------------------------------------------------------------------------------------------------------------------------------------------------------------------------------------------------------------------------------------------------------------------------------------------------------------------------------------------------------------------------------------------------------------------------------------------------------------------------------------------------------------------------------------------------------------------------------------------------------------------------------------------------------------------------------------------------------------------------------------------------------------------------------------------------------------------------------------------------------------------------------------------------------------------------------------------------------------------------------------------------------------------------------------------------------------------------------------------------------------------------------------------------------------------------------------------------------------------------------------------------------------------------------------------------------------------------------------------------------------------------------9923014124899999999987311577777777777777776555115677704788765356653135533331467777777667777751688862016776133115178716884112411130787632888760367777777777448888843677766677777148888605777523022316777776651222467777642212467777777777777666777712477604134589999999999999999998777222677777653400235678887111258999987505667711011113611855899886155552888898851588873116888637888458898844777535654567777777764278999999999999985318886167743677513366776322346764102467787766423223110000126777777778774101789999982124445415665325788876278999999999987124678876428999999311157777777777777777777777777777889---B-B--BBBBBBBB--BBBBBBBB----B----B-B---B--BB-BBBBBBB-------B-BBB-BB-B----BB-B--B------B---B-BBBBBBB-B-B-BB-B-B-B-B----BBB-BB--BB--BB-B-BB--BB--B----B---B-BBBBBBBBBB----B---B-BBBBBBB-----B-BBB--B-BB---BB-B--B---BBB-BBBBBBBBBB----B-------------B-BB--BB--B---B--BBBBBBBBBB------------------BB--B------B--BBB--B--BB--B---BBBB-BBBBBBB-BBBBBBBBB----B--BB-BBB-B-B-BBB-BBBBBB-----BBBBBBBB---BB--BBB---------B-B--BB-BBBBBB-BBBBBB--B---BBBBBBBBBBBBB---B-BBBBBBBBBBBB-B-BB--BBBBBBBBBBBBBBBB--BB---------BBB-BBB-BBB--------B--BBB--B---B---BBB-BB-BBBBBB---B--BB-B--BB-BB--B------B-----B-------------------------------------------B-------------B-------B-BBBBB------------BB---------B--------------------B-B-B-----------------------BB---------B-BB---B-------B-----B-B-B-B--------------BB-BB------B--B--------------------------------------------------------------------------------------------------------------------B--BB--B-------------------BBB-----------B--B-------BB-B-BBB-------BBBB-------B---B---------------B--BB--BB-BB--B---B---BB---B----B-------B-BB-B-BB-B----B---B---BBBBBBBBBB----------------BB--B-------------B--BB---B--------B--BB-BBB-BB---------B--BB--B------------------------------------------------------------------------------------B---------------------------------------------------------------------------BB--------------------------------B-B------------------------------------------------------------------------------------------------------------------------------------------------------------------------------BB------------B-------------B-B-B-------B-B--------------------------------B---B-B---B-------------------------------B------------------BB--B-----------------------B------------------------------B--BB-BB--B-------------B----------------------------------------\*\*\*\*\*\*\*\*\*\*\*\*\*\*\*\*\*\*\*\*\*\*\*\*\*\*\*\*\*\*\*\*\*\*\*\*\*\*\*\*\*\*\*\*\*\*\*\*\*\*\*\*\*\*\*\*\*\*\*\*\*\*\*\*\*\*\*\*\*\*\*\*\*\*
