# Supplementary material for: Towards Understanding Afghanistan Pea Symbiotic Phenotype Through the Molecular Modeling of the Interaction Between LykX-Sym10 Receptor Heterodimer and Nod Factors
Source: Front Plant Sci. 2021 May 7;12:642591. doi: 10.3389/fpls.2021.642591 (PMC8138044; doi:10.3389/fpls.2021.642591)
Supplement: Supplementary file 2 [file Data_Sheet_2.ZIP › MtLYK3.html]

xml version="1.0"?jp\_ZBlhPH5/1-620Lupas\_21Lupas\_14Lupas\_28jnetpredJNETCONFJNETSOL25JNETSOL5JNETSOL0JNETHMMJNETPSSMJNETJURY

xml version="1.0"?102030405060708090100110120130140150160170180190200210220230240250260270280290300310320330340350360370380390400410420430440450460470480490500510520530540550560570580590600610MNLKNGLLLFILFLDCVFFKVESKCVKGCDVALASYYIIPSIQLRNISNFMQSKIVLTNSFDVIMSYNRDVVFDKSGLISYTRINVPFPCECIGGEFLGHVFEYTTKEGDDYDLIANTYYASLTTVELLKKFNSYDPNHIPVKAKINVTVICSCGNSQISKDYGLFVTYPLRSDDTLAKIATKAGLDEGLIQNFNQDANFSIGSGIVFIPGRDQNGHFFPLYSRTGIAKGSAVGIAMAGIFGLLLFVIYIYAKYFQKKEEEKTKLPQTSRAFSTQDASGSAEYETSGSSGHATGSAAGLTGIMVAKSTEFTYQELAKATNNFSLDNKIGQGGFGAVYYAELRGEKTAIKKMDVQASSEFLCELKVLTHVHHLNLVRLIGYCVEGSLFLVYEHIDNGNLGQYLHGIGTEPLPWSSRVQIALDSARGLEYIHEHTVPVYIHRDVKSANILIDKNLRGKVADFGLTKLIEVGNSTLHTRLVGTFGYMPPEYAQYGDVSPKIDVYAFGVVLYELITAKNAVLKTGESVAESKGLVQLFEEALHRMDPLEGLRKLVDPRLKENYPIDSVLKMAQLGRACTRDNPLLRPSMRSIVVALMTLSSPTEDCDDDSSYENQSLINLLSTR------------------------------------------------------------------------------------------------------------------------------------------------------------------------------------------------------------------------------------------------------------------------------------------------------------------------------------------------------------------------------------------------------------------------------------------------------------------------------------------------------------------------------------------------------------------------------------------------------------------------------------------------------------------------------------------------------------------------------------------------------------------------------------------------------------------------------------------------------------------------------------------------------------------------------------------------------------------------------------------------------------------------------------------------------------------------------------------------------------------------------------------------------------------------------------------------------------------------------------------------------------------------------------------------------------------------------------------------------------------------------------------------------------------------------------------------------------------------------------------------------------------------------------------------------------------------------------------------------------------------------------------------------------------------------------------------------------------------------------------------------------------------------------------------------------------------------------------------------------------------------------------------------------------------------------99214148999999988615655567871132333116778770130312046676666662112777777776667777744888861177777531254657862687201213101477612777762367777753577448888605777766777720688986268862323666426640888640146777777771010177777777777777777777710000275024899999998864002667777777777777777777777777777777777777777777777762143688988623677665211157761799985561899984275437899999999986157862577636548855899860577776665677777776428999999999999998526888616774367766566677630124675122367887357777532467764121116656776321122200110102577777777777777213899999988137333088764167888762789999999999873235567775289999986036777777777777765422578899------BB--B--B-BBBB-B-------B-BBBBBBBBB----B-BBB-BB-B-------B--BB-BB--BB-----B----BB-BBBBB-B--B-BBBB-B-B-B----BBB-BB--BB-BBBBB-BB--BB-B----B---B-B-BBB-BBB----B---B-BBBBBBB-----B--BB--B-B---BB--BB--B-BB----BBBBBB-B--B-BB-B-B--------BBBBBBBBBBBBBBBBBBBBBBBB----------------B--------------------------B--B----B--B-B--B--BB--B---BBBB-BBBBBBB-B-B----BBBB-B---B---B--BB-BBB-B-B-BBB-BBBBB----BBBBBBBB---BB--BBB------B-B--BB-BBBBBB-BBBBBB--B---BBBBBBBBBBBBB---B-BBBBBBBBB-BB------BBBBBBBBBBBBBBBBB--B-BB--BBBBBBBBBBBBBBBB--BB-----------BBB-BB--BB--B-----B--BBB--B---B---BBB-BB-BBBBBB---B--BB-B--BB-BB--B------B----------B-----------------------------------B-BBBBB-----------B--B-------------B-------------------B-B-B-B------------------------BB---------B-BB----------B-----B-B-B-B------------BBBBBB------B--B--------------------------------------------------------BB-BB-B--B-----------------------------------------------------------------B--B--BB--------------B-BB-B------BBB--B-------B---B-BB----B--BB-B-BBB-----BBBB-BB----B---B------------B--BB--BB-BB--B-------BB---B----B-------B-BB-BBBB---------B-B-B-BB-BBBBBBB---------B---BBB-BBBBBBBB----------------BB--B--BB--------B--BB---B--------B--BB-BBB-BB---------B--BB--B--------------------------------------------------------B--B-------------------------------B---------------------B------------------------------------------B------------------B-B---------------BB---------------------------------------------------------------------------------------------------------------------------------------------------B------------------------------BBB--------------B---------------B-B------BB---B-------------------------B---B-B---B-----------------BB---------------------------------B-B-------------------BB--B-------------------------B-----------------------------B--BB-BB--B-------------B--------------------------------\*\*\*\*\*\*\*\*\*\*\*\*\*\*\*\*\*\*\*\*\*\*\*\*\*\*\*\*\*\*\*\*\*\*\*\*\*\*\*\*\*\*\*\*\*\*\*\*\*\*\*\*\*\*\*\*\*\*\*\*\*\*\*\*\*\*\*\*\*\*\*\*\*\*\*
