# Supplementary material for: Towards Understanding Afghanistan Pea Symbiotic Phenotype Through the Molecular Modeling of the Interaction Between LykX-Sym10 Receptor Heterodimer and Nod Factors
Source: Front Plant Sci. 2021 May 7;12:642591. doi: 10.3389/fpls.2021.642591 (PMC8138044; doi:10.3389/fpls.2021.642591)
Supplement: Supplementary file 2 [file Data_Sheet_2.ZIP › MtNFP.html]

xml version="1.0"?jp\_Zyvwdlx/1-595Lupas\_21Lupas\_14Lupas\_28jnetpredJNETCONFJNETSOL25JNETSOL5JNETSOL0JNETHMMJNETPSSMJNETJURY

xml version="1.0"?102030405060708090100110120130140150160170180190200210220230240250260270280290300310320330340350360370380390400410420430440450460470480490500510520530540550560570580590MSAFFLPSSSHALFLVLMLFFLTNISAQPLYISETNFTCPVDSPPSCETYVAYRAQSPNFLSLSNISDIFNLSPLRIAKASNIEAEDKKLIPDQLLLVPVTCGCTKNHSFANITYSIKQGDNFFILSITSYQNLTNYLEFKNFNPNLSPTLLPLDTKVSVPLFCKCPSKNQLNKGIKYLITYVWQDNDNVTLVSSKFGASQVEMLAENNHNFTASTNRSVLIPVTSLPKLDQPSSNGRKSSSQNLALIIGISLGSAFFILVLTLSLVYVYCLKMKRLNRSTSSSETADKLLSGVSGYVSKPTMYEIDAIMEGTTNLSDNCKIGESVYKANIDGRVLAVKKIKKDASEELKILQKVNHGNLVKLMGVSSDNDGNCFLVYEYAENGSLEEWLFSESSKTSNSVVSLTWSQRITIAMDVAIGLQYMHEHTYPRIIHRDITTSNILLGSNFKAKIANFGMARTSTNSMMPKIDVFAFGVVLIELLTGKKAMTTKENGEVVILWKDFWKIFDLEGNREERLRKWMDPKLESFYPIDNALSLASLAVNCTADKSLSRPTIAEIVLCLSLLNQPSSEPMLERSLTSGLDAEATHVVTSVIAR------------------------------------------------------------------------------------------------------------------------------------------------------------------------------------------------------------------------------------------------------------------------------------------------------------------------------------------------------------------------------------------------------------------------------------------------------------------------------------------------------------------------------------------------------------------------------------------------------------------------------------------------------------------------------------------------------------------------------------------------------------------------------------------------------------------------------------------------------------------------------------------------------------------------------------------------------------------------cccccccccccccc-------------------------------------------------------------------------------------------------------------------------------------------------------------------------------------------------------------------------------------------------------------------------------------------------------------------------------------------------------------------------------------------------------------------------------------------------------------------------------------------------------------------------------------------------------------------------------------------------------------------------------------------------------------------------------------------------------------------------------------------------------------------------------------------------------------------------------------------------------------------9330033138999999999874413377777777765467777721100388880478876427776213464110000577777766777775068886157777665466317850787401241432127763788876036777777777744888885367776435677102588871367771000002777761132210367777777776021167776777777776677777511011241158999999999999999999998224788763211122267888876122258999987506767760689313870764899974270489999998724788871168886318862788886157777666567777777777777642789999999999999852688861677436775103667762164367777677764677764121243310101267777777787741137899998861477655255543267888762789999999999873246788764289999872157777777777777777777777777777889-----B-B-B--BB--B-BBBBB-B-B---------B-B---B--BB-BBBBBBB-----B-B-BBB-BB-B----BB-----------B---B-B-BBB-B-BB---BBB-B-B-B----BBBBBB--BB--BB-B-BB--BBB-B---BBBB-B-BBBBBBBBBB---BB---B-BBBBBBBB---BB--BB--B-B--B-BB--BB-BB--B--BBBBBBBBBBB-B-BBB--B--------BBBBBBB--B-B-B--BBB-BBBBBBB-----------------B---B--BB--B--BBB--B--BB--B---BBBB-BBBB-BB-B-BBBBB-B---B--BB-BBB-B-B-BBB-BBBBBB-----BBBBBBBB---BB--BBB----------B-B-B--BB-BBBBBB-BBBBBB--B---BBBBBBBB-BBBB---B-BBBBBBBBBBBB-BBBB--BBBBBBBBBBBBBBBB--BB---------BBB-BBB-BBBB-------B--BBB--B---B---BBB-BB-BBBBBB---B---B-B--BB-BB--B------------B--------------B-------------------B--B------------------B-------B-BBBB----------B--BB---------B------------------B-B-B-B----------B---B--------BB---B-----B-BB-----------B-----B-B-B-B--------------BB-B-------B--B-----------B------------------B------------------------BB----------------------------------------------------------B--BB--B-------------------BBB------B---B--B-------BB-B-BBB-------BBBB-B-----B---B------------------BB-BB--BB-BB--B---B---BB---B----B-------B-BB-B-BB------BB--B---BBBBB-BBBB-B--------------BB--B---B---------B--BB---B--------B--BB-BBB-BB---------B--BB--B-----------------------------------------------------------------------------------B-----------------------------------------------B---------------------------B-------------B-------------------B-B--------------------------------------------------------------------------------------------------------------------------------------------------B--------------------------BB----------------------B----B-B---------B-----------------------------------B---B-B---B-----------------B---------B---B------------------BB-BB-----------------------B-----------------B------------B--BB-BB--B-------------B--------------------------------------\*\*\*\*\*\*\*\*\*\*\*\*\*\*\*\*\*\*\*\*\*\*\*\*\*\*\*\*\*\*\*\*\*\*\*\*\*\*\*\*\*\*\*\*\*\*\*\*\*\*\*\*\*\*\*\*\*\*\*\*\*\*\*\*\*\*\*\*\*\*\*\*\*\*\*\*\*\*\*\*\*\*\*\*\*\*\*
